# Ecological brain: reframing the study of human behaviour and cognition

**DOI:** 10.1098/rsos.240762

**Published:** 2024-11-08

**Authors:** Gabriella Vigliocco, Laura Convertino, Sara De Felice, Lara Gregorians, Viktor Kewenig, Marie A. E. Mueller, Sebastijan Veselic, Mirco Musolesi, Andrew Hudson-Smith, Nicholas Tyler, Eirini Flouri, Hugo J. Spiers

**Affiliations:** ^1^ Leverhulme Doctoral Training Programme for the Ecological Study of the Brain, University College London, London, UK; ^2^ Experimental Psychology, University College London, London, UK; ^3^ Institute for Cognitive Neuroscience, University College London, London, UK; ^4^ Division of Psychiatry, University College London, London, UK; ^5^ Institute of Neurology, University College London, London, UK; ^6^ Computer Science, University College London, London, UK; ^7^ Centre for Advanced Spatial Analysis, University College London, London, UK; ^8^ Civil, Environmental and Geomatic Engineering, University College London, London, UK; ^9^ Institute of Education, University College London, London, UK

**Keywords:** ecological validity, human behaviour, brain functions, real-world neuroscience, real-world cognitive science, exploratory science, confirmatory science

## Abstract

The last decade has seen substantial advances in the capacity to record behaviour and neural activity in humans in real-world settings, to simulate real-world situations in laboratory settings and to apply sophisticated analyses to large-scale data. Along with these developments, a growing number of groups has begun to advocate for real-world neuroscience and cognitive science. Here, we review the arguments and the available methods for real-world research and outline an overarching framework that embeds key ideas proposed in the literature integrating them into a cyclic process of ‘bringing the lab to the real world’ (recording behavioural and neural activity in real-world settings) and ‘bringing the real-world to the lab’ (manipulating the environments in which behaviours occur in the laboratory) that combines exploratory and confirmatory research and is interdisciplinary (including those sciences concerned with the natural, built or virtual environment). We highlight the benefits brought by this framework emphasizing the greater potential for novel discovery, theory development and human-centred applications to the environment.

## 1. Brain and behaviour in the real world and in the laboratory

### 1.1. The ecological validity of experiments

The human brain has evolved to survive in complex environments. We engage with and adapt our behaviours to who and what surrounds us. For example, to get from A to B, we may use external aids (e.g. GPS-guidance), or knowledge of the typical location of landmarks (e.g. tills to get out of a supermarket). If a place is particularly crowded, we may ask other people for help. Thus, the way we engage specific cognitive functions (e.g. navigation) depends on the specific environment, namely, the physical as well as social setting in which the brain operates in any given moment (e.g. while walking across a street, or making decisions, or talking to others). Yet, within psychology and cognitive neuroscience, the traditional approach to studying behaviour has involved experiments that do not incorporate the multiple, complex and interrelated variables typical of real-world environments, nor the interplay between different cognitive functions in specific environments. Generally, cognition and behaviour have been mainly investigated within a reductionist approach, broadly defined as the practice of understanding more complex scientific phenomena in terms of smaller component parts. Reductionism prioritizes parcellation of cognitive functions and control of environmental variables, to ensure that the phenomena are easily identifiable and specified to a point that affords the establishment of causal relationships, at the expense of accounting for human behaviour in the specific environments that characterize our daily lives. While this ensures that causal factors can be identified, their impact can be limited to those same conditions identified in the specific experiment which may differ from those in our everyday life.

Over the years many scholars have questioned this way of conducting scientific investigations and there have been various calls for more ecologically valid, real-world approaches to psychological and neuroscientific research. The term ecological validity was first introduced by Brunswik [1,2] to refer to the correlation between a proximal sensory cue (e.g. stimulation of the retina) and a distal variable (e.g. object in the environment). It was then adapted by Orne [3] to refer to the generalization of experimental findings to the real world outside the laboratory (see [4] for a discussion of the relation between these two meanings of the term). Nowadays the term is used in a way more similar to Orne [3], to refer to the extent to which results of a study have a bearing on real-world behaviour, in terms of both generalizability of the findings and their practical implications [3–6]. Ecological validity has been used to refer to the stimuli (i.e. emphasis on the use of more naturalistic materials) and/or the task (i.e. use of more life-like tasks) (see [6] for a more detailed discussion). Generalizability to real-world settings is, however, often an implicit underspecified assumption, rarely explicitly tested in psychology and cognitive neuroscience (see [6] for a discussion). Ecological validity requires consideration of the individual (their body not just the brain, and their history) as well as consideration of the specific environments in which behaviours take place [7]. Even when the behavioural and neural mechanisms investigated in the laboratory and those in the real-world situations are highly similar, still insufficient consideration of environmental elements can lead to faulty inferences and ill-posed questions/predictions about how humans behave as different environments may determine different behaviours, depending on what would be the most suitable [8] or ‘optimal’ [9] behaviour. As Kihlstrom [10] put it: ‘The purpose of laboratory research is to understand the real-world: to make the problem simple so that it can be studied effectively, and to control relevant variables so that important relations, especially causal relations, can be revealed. Unfortunately, generalisation from the lab to the real-world requires an inferential leap: its legitimacy depends on the degree of similarity between the conditions that are obtained in the laboratory and those found in the real-world’ (p. 6).

Especially in recent years, thanks to the advent of new technologies that allow for mobile recording of behaviour and brain activity simultaneously, from multiple agents, and in the wild, as well as the availability of new analytical tools [11–14], many scholars have raised concerns relating to the ecological validity of experiments carried out within our traditional reductionist approach and have made proposals to improve ecological validity and therefore generalizability from behavioural and neural responses elicited in the laboratory to those elicited in real-world situations [3,15–29]. In this paper, we first briefly summarize the proposals that have been put forward to increase the ecological validity of studies and the methods that can be used to carry out real-world cognitive science and neuroscience. We then conclude by presenting a framework that integrates these previous proposals in a broader approach to the study of humans in their environment.

## 2. Towards real-world cognitive science and neuroscience

Bannister [30] described the traditional reductionist approach to human research in the following manner: ‘In order to behave like scientists, (experimental psychologists) must construct situations in which our subjects are totally controlled, manipulated and measured. We must cut our subjects down to size. We construct situations in which they can behave as little like human beings as possible and we do this in order to allow ourselves to make statements about the nature of their humanity’.

A number of recent papers have discussed the main limitations of traditional experimental paradigms and provided solutions to improve ecological validity. Two key criticisms raised to the traditional reductionist experimental approach are (i) the use of impoverished rather than multidimensional and dynamic stimuli (e.g. using static pictures to study emotion processing) and (ii) the use of artificial and decontextualized rather than real-life-like tasks (e.g. asking participants to decide if a string of letters is a word to study language processing) that force participants to ‘behave as little as possible like human beings’ as Bannister put it [30].

The need to move from impoverished, often unidimensional and static stimuli to the use of real-life-like paradigms involving complex and dynamic stimuli has been argued for by a number of scholars [27,28]. For example, pictures are being widely used in research as proxy of real objects; however, our processing of images differs from our processing of real objects in many important ways, most prominently the fact that images do not afford actual actions. Images may evoke actions, but they lack actability, the potential to interact with the represented object meaningfully [27]. To study real-life sensory experience, neuroscientists are increasingly employing dynamic videos, speech and music that incorporate sensory stimuli typically encountered in everyday life [31,32]. These paradigms provide a reasonable approximation to how we encounter stimuli in everyday life and therefore are preferable to the controlled and impoverished stimuli traditionally used. As reviewed by Sonkusare *et al.* [28], evidence suggests that the brain may be more strongly ‘tuned’ to naturalistic than artificial stimuli. Life-like stimuli have been shown to lead to quantitative changes in responses, including improvements in memory [26,33,34], object recognition [35], attention and gaze capture including in infants [36,37]. These findings show how realistic stimuli can amplify or strengthen behavioural and brain responses that might otherwise be difficult to observe when relying on proxies. Thus, increasing the naturalness of the stimuli used in experiments is one possible way in which ecological validity of experiments can be improved.

The use of life-like tasks that do not limit the active role of participants is another way in which researchers have improved the ability of experiments to generalize to real-world situations [23,26]. The importance of considering participants as active agents has been strongly argued for in social neuroscience [38,39]. For decades in this field, the ‘social’ component of social cognition research was limited to individuals observing a social situation while sitting alone in front of a computer screen or constrained in an fMRI scanner. While this provides a clear paradigm within which to test hypotheses, it does not necessarily adhere to how humans process and experience social interactions in real life leading to questioning the value of such an approach. In recent years, ‘second-person’ neuroscience has become increasingly popular [38,39]. Its basis is the assumption that observing social interactions is fundamentally different from engaging in social interactions, from either a neurobiological, physiological, cognitive or behavioural perspective [38]. For example, it has been shown that key regions of the ‘mentalizing network’ (including the ventral medial prefrontal cortex (vmPFC), dorsomedial prefrontal cortex (dmPFC) and temporoparietal junction (TPJ)) do not respond selectively only to tasks (with solo participants) requiring thinking about another person’s mental states, but are consistently found to be sensitive to level of engagement with a social partner, regardless of task demands [40–45].

Naturalistic paradigms can address questions concerning brain and behavioural functions in the real world, therefore allowing us to ‘bring the laboratory to the real world’. This approach leverages mobile recording (see [46] and table 1*a*) to improve ecological validity by investigating brain and behaviour directly in the real world, thus allowing researchers to study, for example, the brain dynamics underscoring learning of new material in the classroom by pupils [46,70]. It overcomes the two limitations described above; it does introduce, however, a very large degree of complexity where true causes of behaviour are difficult to disentangle from other sources of variability. A complementary approach consists of ‘bringing the real world to the laboratory’. Here, some degree of control on the stimuli and setting is used in the context of non-reductionist paradigms. One example is to ask participants to watch full-length movies, or listen to audio books while they are scanned to investigate language comprehension or semantic processing [31], or asking participants to navigate simulated real-world spaces while in the scanner [71]. An important issue with this approach is to know which dimensions of the real-world experience can be controlled or eliminated, given that our hypotheses are most often based on prior experimental (controlled) studies. Kihlstrom [4] further cautions that: ‘An experiment can employ extremely life-like stimulus materials in an extremely lifelike setting… But the experiment would still lack ecological validity if it also contained demand characteristics that are not present in the nonexperimental situation that it is intended to represent’ (pp. 468–469).

**Table 1.** A survey of methods for real-world cognitive science and neuroscience.

method	description	key reference
**(*a*) methods that allow to bring the laboratory to the real world**		
(mobile) electroencephalography (EEG)	non-invasive recording of electrical activity of the brain	‘Mobile EEG in research on neurodevelopmental disorders: opportunities and challenges’ [47]
(mobile) functional near-infrared spectroscopy (fNIRS)	non-invasive recording of brain activity detecting changes in blood flow	‘A review on the use of wearable functional near-infrared spectroscopy in naturalistic environments’ [48]
(mobile) optically pumped magnetoencephalography (op-MEG)	non-invasive recording of brain activity detecting magnetic fields produced by the brain’s electrical currents	‘Moving magnetoencephalography towards real-world applications with a wearable system’ [49]
global positioning system (GPS)	provides (real-time) geolocation and time information	‘Review of GPS travel survey and GPS data-processing methods’ [50]
indoor tracking	provides (real-time) geolocation through tracking devices (e.g. Bluetooth)	‘Indoor tracking: theory, methods and technologies’ [51]
(mobile) electrodermal activity (EDA)/galvanic skin response (GSR)	monitoring changes in the skin’s electrical conductance, due to sweat production	‘Neighbourhood environments influence emotion and physiological reactivity’ [52]
heart rate (HR)/heart rate variability (HRV)	monitoring average heart beats and variability between heart beats	‘Interoceptive ability predicts survival on a London trading floor’ [53]
(mobile) eye-tracking	monitoring real-time changes of eye gaze direction and duration	‘Head-mounted eye tracking: a new method to describe infant looking’ [54]
mobile sensing using smartphones	monitoring and extraction of a variety of information using sensors that are embedded in mobile phones	‘The rise of people-centric sensing’ [55]
**(*b*) methods that allow to bring the real world to the laboratory**		
virtual reality (VR)	performing tasks in computer-generated environments	‘Can simulated nature support mental health? Comparing short, single-doses of 360 degree nature videos in virtual reality with the outdoors’ [56]
augmented reality (AR)	overlaying computer-generated aids onto real environments	‘Is that me?—embodiment and body perception with an AR mirror’ [57]
manipulated and controlled physical environments	reconstructing full-scale environments to minimize real-world unpredictability and enable experimental control	‘Train design features affecting boarding and alighting of passengers’ [58]
**(*c*) data processing and modelling of real-world data**		
space syntax	theory and method for investigating relationships between society and space	‘Ward layout, communication and care quality: spatial intelligibility as a key component of hospital design’ [59]
geographic information system (GIS)	a spatial system to create, manage, analyse and map location data (where) and attribute data (what)	‘Geo-EEG: towards the use of EEG in the study of urban behaviour’ [60]
facial expression and body-pose estimation	automatic facial behaviour analysis toolkit with available source code for both running and training the models OpenPose is a popular computer vision real-time system designed for multi-person keypoint detection. It can identify and track various human body parts, including the body, foot, face and hands, through images and videos	‘OpenFace 2.0: facial behavior analysis toolkit’ [61]; OpenPose [62]
longitudinal and cohort studies	collecting multi-purpose data on a large sample to investigate relations between outcomes (e.g. health) and exposures (e.g. deprivation), often over time, in the general population and in subpopulations	‘The role of neighbourhood greenspace in children’s spatial working memory’ [63]
encoding and decoding of fMRI data	*encoding*: by fitting large feature vectors to voxel-wise fMRI activity, it is possible to account for a wide range of environmental variables and complex, nonlinear interaction effects typical of, e.g. movie watching. *Decoding*: reconstruction of visual, semantic or other information from non-invasive brain recordings (MEG, fMRI, EEG)	*encoding*: ‘Natural speech reveals the semantic maps that tile human cerebral cortex’ [64]; ‘The revolution will not be controlled: natural stimuli in speech neuroscience’ [65]*. decoding*: ‘Semantic reconstruction of continuous language from non-invasive brain recordings’ [66]; ‘I can see what you see’ [67]
data-driven agent-based models	by using data from real-world experiments, it is possible to build models describing the behaviour of individuals, groups, communities and cities	‘An introduction to agent-based modeling: modeling natural, social and engineered complex systems with NetLogo’ [68]
foundational model-based simulations and experiments	the advent of foundational models is opening new possibilities for studying behaviour and interactions in simulated environments, by exploiting their richness and expressivity	‘Generative agents: interactive simulacra of human behavior’ [69]

A proposed solution to this issue is to relax the divide between data-driven and hypothesis-testing approaches as argued by, for instance, the ‘system identification approach’ in cognitive neuroscience [23,72]. Here, hypotheses are not formulated in terms of which predetermined dimensions of stimuli should engage specific neural systems, as in the traditional reductionist approach, but in terms of how well a number of alternative explicit computational models, trained to encode an observed pattern of neural responses from a subset of the stimuli, predict neural responses to new stimuli (validation set) using large random samples of naturalistic stimuli such as audio books [73]. These inductive methods have been argued to provide a better approximation of how naturalistic stimuli map into brain networks [72,74]. However, results from this approach tend to be difficult to interpret [75] and while it may work well with fMRI data it is not clear to what extent it could be used more broadly across cognitive domains and behavioural responses.

A broader approach, proposed by Matusz *et al*. [76] and before that by Kingstone [77], is in terms of a research cycle. Their framework goes beyond calling for the use of naturalistic stimuli and life-like settings to proposing how these can be integrated into a research cycle. In particular, they envision three stages: (i) classic laboratory research; (ii) naturalistic laboratory research (similar to what we referred to above as bringing the real world to the laboratory); and (iii) fully naturalistic research (similar to what we referred to as the approach of bringing the laboratory to the real world). They portray these three stages as complementary rather than alternative manners to approach cognitive neuroscience questions highlighting the importance and complementarity of both confirmatory (hypothesis-driven, typical of classic laboratory experiments) but also exploratory (data-driven, typical of fully naturalistic research) research in the scientific process. We will return to this framework below when we discuss our own proposal.

In the next section, we review the methods available to researchers for implementing naturalistic paradigms. In addition to methods for recording brain and behavioural functions in the real world and methods for simulating real-world conditions in laboratory settings, we further review methods for data processing and modelling of real-world data.

## 3. Methods available for real-world cognitive science and neuroscience

As discussed above, arguments for more ecologically valid approaches have been laid out throughout past decades [1,2,24,78]. This poses the question of why practices have not yet fully changed. The answer is likely to be because it is very challenging. First, it requires resources (technologies, facilities and interdisciplinary teams) that are not as easily available, especially in research cultures that favour individual contributions over team efforts. Second, it is the case that studies providing better approximation of real-world settings can involve complex designs and produce multidimensional data that are hard to analyse and interpret. Below we summarize a number of methods that can support ecologically valid research.

### 3.1. Taking the laboratory into the real world

**Figure 1.**
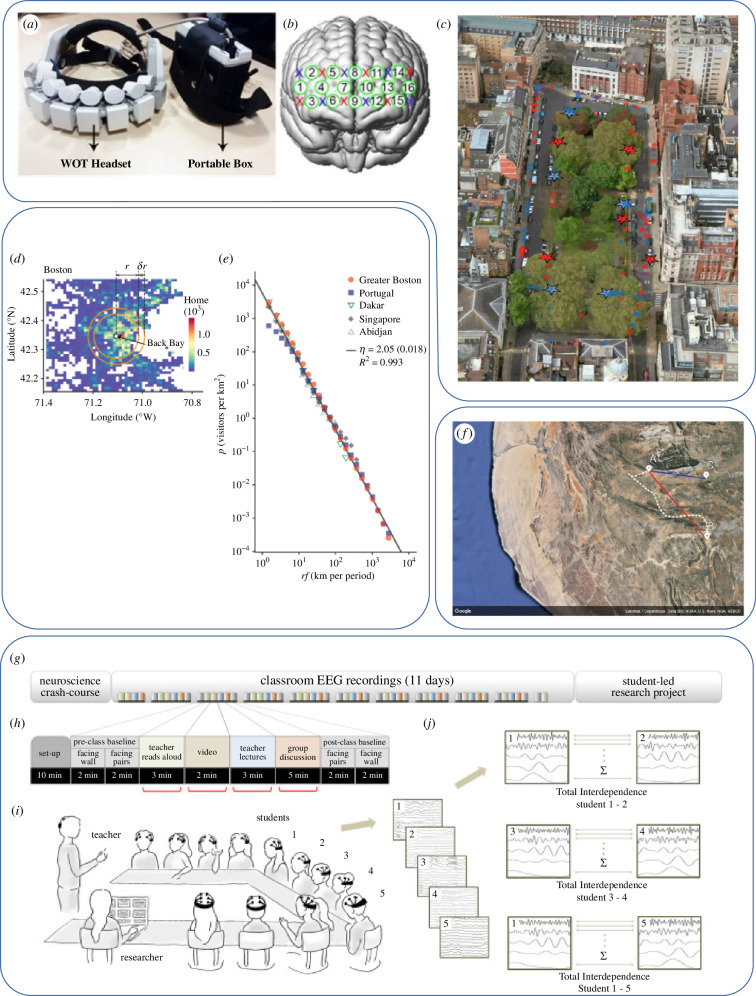
Taking the laboratory into the real world. (*a–c*) Adapted from Burgess *et al.* [79]. Functional near-infrared spectroscopy (fNIRS) was used to record activity from participants walking in Queen Square in London. (*a*) Recording device. (*b*) Sensor locations on the scalp to record prefrontal activity beneath. (*c*) Map of Queen Square marking locations of social prospective memory cues (large blue stars), non-social prospective memory cues (large red stars), social functional haemodynamic events (blue asterisks) and non-social functional haemodynamic events (red asterisks). (*d–f*) Adapted from Schläpfer *et al.* [80]. Large-scale mobility data from across the world are used to develop a scaling law that considers temporal and spatial dimensions of human movement. (*d*) Map indicating population density in Black Bay, Boston, as deduced from mobile phone data. (*e*) Visitor flow is shown to depend on travel distance (*r*) and visiting frequency (*f*), with this scaling relation holding true for different urban regions around the world. (*f*) Adapted from Davis *et al.* [81]*.* Testing large-scale spatial ability; participants at location A point towards locations B and C, or imagine they are at location B and point to location C. (*g–j*) Adapted from Dikker *et al.* [82]. EEG was used to record electrophysiological activity while students were engaged in classroom activities to explore brain-to-brain group synchrony and class engagement. (*g,h*) Experimental set-up involved recording EEG activity of 12 students over 11 teaching days; red bars indicate individual EEG recording sessions. (*i,j*) Twelve students wear EEG headsets to allow for brain-to-brain synchrony to be recorded. All images are combined and reproduced with permission under the Creative Commons Attribution 4.0 International Licence.

Taking the laboratory into the real world to observe phenomena as they naturally unfold can often call for different or novel protocols and methods (see figure 1 and table 1*a*). Researchers may need to follow their participants’ actions in their natural environments, resulting in experimental sessions run in uncommon settings and/or at uncommon hours. Examples include studies where the ‘laboratory’ moved to people’s bedrooms to capture their thoughts after being woken at night [83], to the streets of London where people’s memory and brain activity were measured while they walked around [84], tracked through GPS to locations to test navigation [85] or a classroom where student–teacher interactions were observed and neural synchronization studied to capture learning as it occurred [82]. Mobile brain and behavioural monitoring devices allow the flexibility required in these studies and rapid technical developments may allow even more ambitious paradigms to be developed [86,87]. Mobile smartphone devices with a plethora of sensors also provide excellent opportunities such as the opportunity of on-device computation, including the implementation of machine learning algorithms for behaviour inference [88–90].

### 3.2. Taking the real world into the laboratory

**Figure 2.**
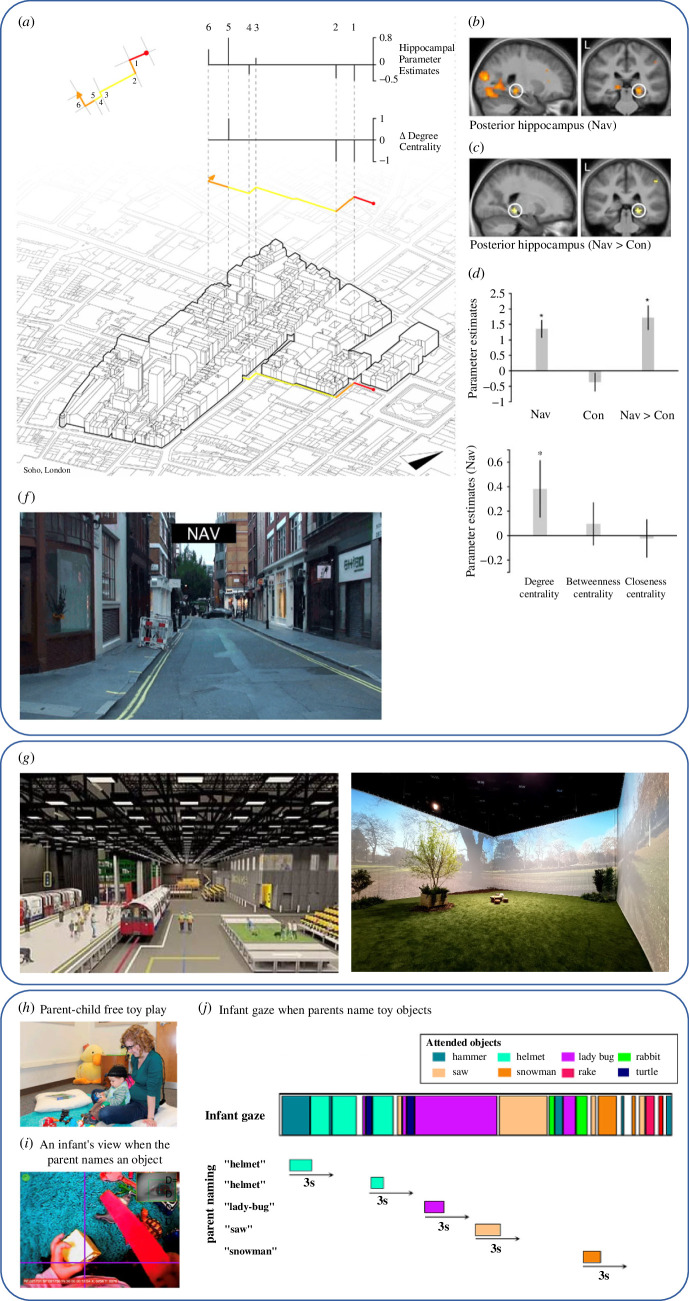
Taking the real world into the laboratory. (*a–f*) Adapted from Javadi *et al.* [71] and Gregorians & Spiers [93]. Graph theoretic analysis has been applied to street networks following a ‘space syntax’ approach based on architecture and urban data science. This analytical approach was combined with an fMRI BOLD data and film simulation of streets of Soho in London (UK) to examine how the brain tracks spatial information during navigation. (*a*) *Top left*: degree centrality plotted for each street segment. *Bottom*: axonometric projection of the buildings on a map of Soho, with degree centrality projected above. *Top right*: change in degree centrality and right posterior hippocampus response at each individual boundary transition (1–6). (*b,c*) During Street Entry Events, right posterior hippocampal activity correlated significantly with the change in degree centrality for Navigation and Navigation > Control. (*d*) Parameter estimates in Navigation and Control conditions for mean activity in the right posterior hippocampus region of interest (ROI) for a model of degree centrality. Error bars denote the s.e.m. (*e*) Parameter estimates in the Navigation > Control condition for mean activity in the right posterior hippocampus ROI for a model containing degree centrality, betweenness centrality and closeness centrality. (*f*) A still from one of the movies used for the fMRI. (*g*) Images reproduced from https://www.pearl.place. Real-world environments are physically recreated at the PEARL facility to study tube carriages and greenspace respectively under controlled conditions. (*h–j*) Adapted from Yu *et al.* [94]. Head-mounted cameras are used to study the link between infant’s attention and caregiver’s naming of objects in home-like environments. (*h*) Infants wear a head-mounted camera while playing with toys freely with their parents. (*i*) Example of an infant’s gaze on an object when the parent names the object. (*j*) Infant gaze aligning with parent’s naming of objects, using a window of 3 s from the onset of the object naming. Images combined and reproduced with permission under the Creative Commons Attribution 4.0 International Licence.

Taking the complexity of the real world to the laboratory allows for the manipulation of environmental variables to establish their causal role. Virtual reality (VR) and augmented reality (AR) enable people to experience highly controlled real-world-like environments at a low cost. Various studies have shown that findings in virtual worlds translate to findings in reality, making these methods particularly useful in terms of ensuring experimental control and ecological validity, while minimizing costs and physical barriers to participating [91]. Theatre-like research facilities can also provide remarkable real-world approximation, allowing us to reconstruct and adapt full-scale physical environments with a high level of precision and control (see UCL’s Person Environment Activity Research Laboratory, PEARL [92]). In figure 2, we present examples of studies that reproduce in the laboratory key elements of complexity from the real world. Table 1*b* provides some of the methods for (re-)creating, controlling and manipulating environments in the laboratory—both digitally and physically.

### 3.3. Analysing and modelling real-world data

The data collected with the methods above are also more complex than in standard laboratory-based experiments, e.g. the use of mobile technologies to carry out research in the real world does not guarantee that they necessarily increase their validity [95]. For example, during real-world wayfinding or navigation experiments, the researcher can control the start and end point of the route that a participant takes, and the task. Much more difficult to control are the unforeseeable events that occur en route, e.g. cars, people, accidents, bad weather. Such variables can affect performance and thus need to be factored into the analysis. Mobile and personal technologies allow for collecting contextual multi-modal data (including social media data) describing the environments in which individuals act [90]. From large longitudinal and cohort studies to studies collecting data in real time, data analytics can be used to explore how people, things and places connect on a much larger scale, allowing for predictions of human behaviour that take into account the many concurring variables (see table 1*c*).

A unique challenge for data analysis posed by brain recordings during naturalistic tasks (e.g. movie-watching) is the presence of linearly and nonlinearly correlated confounding variables. These limit the effectiveness of standard statistical tools such as *t*-tests. However, encoding models may present the solution to this problem: by fitting individual regression models to each voxel, confounding variables may be entangled from the observed effect (if the variable can be quantified and included in an arbitrarily large feature vector [65]). An alternative use of encoding models is to fit word embeddings from LLMs to the activity of each voxel during a given task, to understand how closely LLMs and humans align during, for example, semantic processing [64]. Finally, combining this approach of fitting voxel-wise encoding models with the predictive power of LLMs, it has become possible to reconstruct semantic representations in the human brain from non-invasive brain recordings such as fMRI [66].

Real-world data can also be used to develop a variety of data-driven agent-based models [68], which can be extended to represent complex models of multi-agent systems for studying groups, communities or cities [96]. These *in silico* simulations can be seen as a starting point to develop more concrete hypotheses from real-world patterns that can then be tested in laboratory studies. An example is decision-making, which can be studied using simulated agents as a starting point for experiments involving humans [97,98]. The recent advent of large language models/foundational models [99] is introducing new opportunities in terms of realistic simulation of human behaviour. For example, it is possible to recreate entire simulated societies of agents based on foundational models and observe their interactions and evolution [69]. These studies can be used to develop hypotheses and/or to complement real-world field experiments.

## 4. The ecological brain framework

### 4.1. A cyclic process through ‘the wild’ and ‘the laboratory’

The methods just described provide a toolkit to measure real-life brain and behavioural functions that has the potential to provide powerful insights. Below, we argue methods that ‘bring the laboratory to the real world’ (table 1*a*) and those that ‘bring the real world to the laboratory’ (table 1*b*) should be used in a complementary manner in a cyclic process. We label this framework ‘Ecological Brain’. It brings together different perspectives reviewed above [17,19–21,23,25,26,28,29] providing a general basis for theory building and discovery where research in the real word and in the lab are integrated.

**Figure 3.**
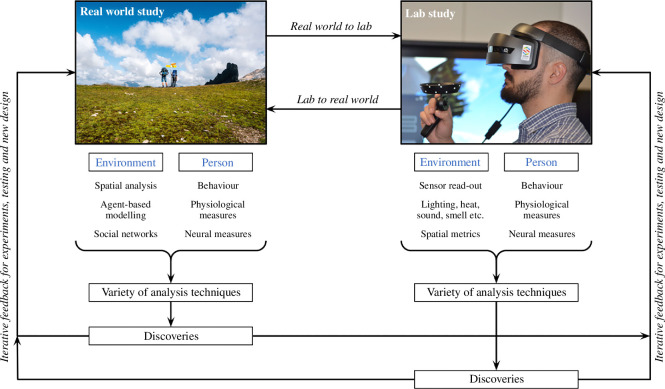
A framework for the study of human brain and behaviour in its ecology. Traditionally, research in ‘the wild’ is considered exploratory and descriptive with marginal bearing on theory development. Research in ‘the laboratory’ is considered as confirmatory and capable of testing theories. In laboratory research, stimuli are simplified and controlled, so the effects can be isolated to the particular aspects of the stimuli and/or task. Here, we illustrate how an ‘Ecobrain’ approach moves beyond this distinction to a cyclic research process in which real-world phenomena are identified and studied both in the real world (*left*) and in the laboratory (*right*). Real-world research, with the use of mobile technologies for continuous recording of behavioural, physiological and neural activity, combined with data-driven modelling approaches, allows for identification of key environmental variables and for prediction of behavioural, physiological and neural responses. The laboratory, supplemented with technologies that allow for control of environmental variables, allows for theory testing in settings that embed key environmental variables identified in real-world research. Laboratory research can also provide new insights that can guide further research in the real world. Image credits: *Top left composite*: EEG image reproduced with permission from Mentalab, contributors to making data acquisition in real-life scenarios possible. Seoul’s skygarden image, reproduced with permission from Ossip van Duivenbode. *Top right composite*: laboratory-based London Underground study image reproduced with permission from PEARL. All other images are combined and reproduced with permission under the Creative Commons Attribution 4.0 International Licence.

Ecological Brain synthesizes, in a general framework, recent real-world approaches to capturing the complexity of human behaviour in interaction with the environment. It proposes a cyclic approach in which exploratory research in the real world provides hypotheses to be tested and guides the design of laboratory experiments, particularly with respect to characterizing environmental variables, but equally, hypotheses that are developed in the laboratory are validated in the real world [23]. This is extended in a continuous cycle of refinements and new hypothesis generation, leading to a new standard in research that cannot be achieved using real-word or laboratory studies alone, regardless of how life-like they are (see figure 3). New (and emerging) technologies and data analytics are key to this approach allowing for a transfer of the rigour of laboratory studies into research in the wild (the real world) and the complexity of the real world into research in the laboratory in such a way that they can inform each other (figure 3). This framework relates to the three-stage cyclical model for real-world cognitive neuroscience research proposed by Matusz *et al.* [76]. Just like Matusz *et al.*, we go beyond calling for the use of naturalistic stimuli and life-like settings to proposing how this can be integrated into a research cycle encompassing both real-world settings and laboratory. We emphasize the importance and complementarity of both confirmatory (hypothesis-driven) and exploratory (data-driven) research in the discovery process, as further discussed below. Our proposal differs from Matusz *et al.*’s one in that we do not see a strong motivation for maintaining traditional (reductionist) laboratory approaches in order to be able to carry out confirmatory research as control of key environmental factors can be achieved in a laboratory setting without necessarily having to sacrifice key elements of environmental complexity. In the Ecological Brain framework, this can be achieved because key environmental factors to manipulate or control in the laboratory are identified in more exploratory studies in the real world (see table 1 and figure 1). Thus, this framework fully brings exploratory and confirmatory research together. More generally, as already argued long ago by others [5,100], the Ecological Brain framework proposes to shift the emphasis from the study of the individual to the study of the interaction between the individual and the environment highlighting the importance of characterizing the environment (being physical or social). Thus, interdisciplinarity is central to the approach. We elaborate on these two aspects below.

### 4.2. The importance of exploratory research for characterizing the environment

Natural behaviours are necessarily contingent on their context. In biological terms, following Gomez-Marin & Ghazanfar [7], context can be conceived as the ‘space’ for the behaviour, given by the interaction between the body of the animal and the physical world around. Each person then may experience the world around differently given differences in their body and their histories (developmental, social and cultural). A key role for the body and for individual differences (within and between groups) have long been recognized [101–104] and are now considered as an important part of our studies and theories [105–107]. However, the interactions of these factors with the specific environments in which the brain operates need also to be considered in the theories that we develop and in the studies testing them. In order to achieve that, the environments need to be characterized, namely, those environmental elements associated with the specific behaviours of interest need to be identified. Examples of how this can be done successfully are provided in figure 1*a*–*f*.

In Ecological Brain, we suggest that naturalistic studies in which people navigate, learn, communicate, etc., in their real-world environments can allow us to identify those key environmental elements. Naturalistic studies therefore inform the experimental investigations in the laboratory. Thus, in the Ecological Brain framework, the environment is not just the stage where our actors (experimental variables) play, but becomes an integral component of the performance. Therefore, operationalizations of the research question(s), hypotheses and predictions have to include aspects of the physical, social, personal and cultural environment [108].

This can be achieved by integrating research ‘in the wild’ and ‘in the laboratory’. Research ‘in the wild’ is more exploratory and, as such traditionally has been considered useful for pilot studies, but overall less important than confirmatory hypothesis-driven research. This is not the case in the Ecological Brain framework where exploratory research is necessary to identify the key contextual variables and therefore it is a necessary step for theory development, leading to hypotheses concerning how these variables or their interactions would affect the phenomenon that can then be experimentally manipulated. Although conclusions drawn from exploratory research cannot identify causes and effects, exploratory ‘in-the-wild’ research using mobile technology and data-driven statistical approaches grounds our studies in the laboratory and therefore it should be treated au par and complementary, with confirmatory laboratory-based research. Moreover, in a cyclic manner, research in the real world can and has been used to test hypotheses and theories developed in the laboratory [109] using computational models to provide theory-driven analyses of large and complex real-world datasets [31,110].

### 4.3. The importance of interdisciplinarity

To be able to meaningfully bring the real world to the laboratory (and vice versa), the environment needs to be characterized and its complexity analysed and related to brain and behaviour responses. For this to happen, psychologists and neuroscientists are not enough. Real-world research must draw from different disciplines: those concerned with methodological and technological advances (e.g. computer science and medical physics) but crucially also, those concerned with the study and quantification of the environment (e.g. engineering, architecture, planning, geography). Thus, the approach advocated here is necessarily interdisciplinary. It does differ, however, from other related approaches (including human ecology, behavioural ecology, environmental psychology, social geography, biological anthropology, population ecology, sociology and neuroethology) in that its interdisciplinarity specifically serves the objective of capturing the complexities of human brain-behaviour, real-world environments and their *bidirectional* relationship. In other words, interdisciplinarity contributes to understanding how humans impact and are impacted by the surrounding environment, including the neural mechanisms engaged in this process. For example, a recent study testing how the environment impacts the development of spatial navigation ability required psychologists to develop a valid test of navigation, geographers to develop new methods to quantify the structure of the environment and computer scientists to apply agent-based modelling to make predictions about behaviour [111]. This means that real-world research should go beyond understanding behaviour and its underlying brain functions *per se*. These contributions are *bidirectional*, as it is the case not only that insights from other disciplines are necessary to better understand brain and behaviour, but also that a better understanding of brain and behaviour can lead to novel insights in the other fields.

## 5. Concluding remarks

A growing number of cognitive scientists and neuroscientists have argued that understanding brain, behaviour and their relationship requires understanding how people function in their ecology [17,19–21,23,25,26,28,29]. While we believe that reductionism has served psychology and cognitive neuroscience well as a principle for scientific research, adherence to pure reductionism has produced fragmented research where parcellization of mental events is most often linked to empirical tractability in the lab, rather than a deeper understanding of mental functions in their ecology [112,113].

The environment, the context traditionally controlled in laboratory studies, cannot be dismissed *a priori*. We go beyond calling for more naturalistic stimuli and life-like tasks in experiments to proposing a continuous cycle between research in the real world and in the laboratory combining: (i) mobile methods that allow for more exploratory studies designed to characterize brain and behavioural responses in specific real-world contexts and (ii) VR, AR and other methods that allow for bringing those complex environmental variables (identified in real-world studies) in controlled laboratory settings. In the Ecological Brain approach, new discoveries come about as a consequence of the iteration between real-world and laboratory-based research (figure 3).

Shifting how we study brain and behaviour can change how we describe/operationalize our object of investigation. For example, after Gibson [16,36] introduced the concept of affordances (natural (i.e. not learnt) relationships between perceptual properties of the environment and relevant actions), the ways in which scientists thought about vision was expanded. Instead of considering vision simply as a process of classification or categorization, introducing affordances made vision an active process in which different visual stimuli carry different weights depending upon what they afford a person to do. We suspect shifting the focus of studying phenomena through the cyclic framework proposed here can lead to questioning at least some existing tenets in the study of human cognition. In this way, the Ecological Brain framework may help the field to move beyond the current criticisms concerning methodological practices [114,115], and lack of clear theoretical models [116].

It is important to acknowledge that conducting research that can be open-ended, data-driven, with multi-level data collection either outside the laboratory or in the laboratory with close-to-real-world conditions, is still very challenging. There are however ways in which the scientific community can support a broader uptake of this approach. One way is by sharing preprocessed data acquired in the real world (including code for processing and analysis as well as tools for reproducibility and for enabling further experiments). For example, the Naturalistic Neuroimaging Database (https://www.naturalistic-neuroimaging-database.org/ [32]) provides fMRI data from 86 participants each watching a full-length movie that can be used to address a variety of questions (e.g. anxiety and amygdala connectivity [117]). Datasets containing mobile sensing data of entire populations of individuals are also increasingly available. Examples include Reality Mining at MIT (https://hd.media.mit.edu/reality/reality/) [118] and StudentLife at Dartmouth (https://studentlife.cs.dartmouth.edu/) [119]. We strongly encourage others to make naturalistic datasets available as well as any code, and analytical tools (e.g. https://naturalistic-data.org/content/intro.html [120]) such that we can move closer to unravel the mysteries of the human brain and behaviour in its ecology.

## Data Availability

This article has no additional data.
